# The Death Penalty

**Published:** 1889-04

**Authors:** 


### HALL'S JOURNAL OF HEALTH

TRUTH DEMANDS NO SACRIFICE: ERROR CAN MAKE NONE.

VOL. 36. APRIL, 1889 No. 4.

### THE DEATH PENALTY.

It is a very grave question whether the taking of human life under the forms of law, as a forfeit for the worst offences, is ever justifiable. That it has been practiced from time immemorial, furnishes no criterian to the present age of progress and enlightenment. At a period, not so far distant as to have faded out of mind, many trivial offences were so punished. Sir William Blackstone in his Commentaries on the laws of England, enumerates 160 distinct crimes to which the death penalty attached, some of the lesser being stealing goods in a shop to the value of five shillings, and couterfeiting the stamps for the sale of perfumery or hair powder.

In all newly settled countries, where the administration of justice is attended with difficulties, owing to the wide extent of judicial districts, and the infrequency of sessions, there is always a tendency on the part of the inhabitants, to take the law into their own hands, and make sure of ridding themselves of the evil doer in a summary way, which, in the fever of their resentment and comparative insecurity, seems less cruel, than the more deliberate action of organized tribunals.

But in later years the tendency has been of a nature more humane, until now, in most civilized countries, the death penalty is confined practically to murder and treason.

In some few of the United States the experiment has been made of abolishing the death penalty altogether, and in three or four states as in some of the Swiss Cantons, after abolition, it has been restored, as more conducive to the security of human life.

The modes of execution practiced by civilized nations are for the most part confined to three, viz: strangulation by hanging; by the garrotte, and by decapitation.

In the state of New York all criminals convicted of a capital offence, committed after the beginning of the present year, and condemned to be executed, are to suffer the penalty by an electrical process which is expected to be instantaneous and effectual.

In view of this measure, some very interesting experiments have recently been made,under the direction of an authorized commission in the course of which certain animals were subjected to the destroying fluid, with results so satisfactory that it is believed that by the adoption of the New York system, the pains and horrors of executions will be largely abated and the brutalizing spectacle of a public execution, a thing of the past.

The next step in the advance should be the permanent abolition of the death penalty which has many sincere advocates among those who have considered the subject in all its bearings.

